# An Overview on the Differential Interplay Among Neurons–Astrocytes–Microglia in CA1 and CA3 Hippocampus in Hypoxia/Ischemia

**DOI:** 10.3389/fncel.2020.585833

**Published:** 2020-11-11

**Authors:** Daniele Lana, Filippo Ugolini, Maria G. Giovannini

**Affiliations:** ^1^Department of Health Sciences, Section of Clinical Pharmacology and Oncology, University of Florence, Florence, Italy; ^2^Department of Health Sciences, Section of Anatomopathology, University of Florence, Florence, Italy,

**Keywords:** CA1 hippocampus, CA3 hippocampus, triads, confocal microscopy, brain metabolism, neurovascular unit, glymphatic system, clasmatodendrosis

## Abstract

Neurons have been long regarded as the basic functional cells of the brain, whereas astrocytes and microglia have been regarded only as elements of support. However, proper intercommunication among neurons–astrocytes–microglia is of fundamental importance for the functional organization of the brain. Perturbation in the regulation of brain energy metabolism not only in neurons but also in astrocytes and microglia may be one of the pathophysiological mechanisms of neurodegeneration, especially in hypoxia/ischemia. Glial activation has long been considered detrimental for survival of neurons, but recently it appears that glial responses to an insult are not equal but vary in different brain areas. In this review, we first take into consideration the modifications of the vascular unit of the glymphatic system and glial metabolism in hypoxic conditions. Using the method of triple-labeling fluorescent immunohistochemistry coupled with confocal microscopy (TIC), we recently studied the interplay among neurons, astrocytes, and microglia in chronic brain hypoperfusion. We evaluated the quantitative and morpho-functional alterations of the neuron–astrocyte–microglia triads comparing the hippocampal CA1 area, more vulnerable to ischemia, to the CA3 area, less vulnerable. In these contiguous and interconnected areas, in the same experimental hypoxic conditions, astrocytes and microglia show differential, finely regulated, region-specific reactivities. In both areas, astrocytes and microglia form triad clusters with apoptotic, degenerating neurons. In the neuron–astrocyte–microglia triads, the cell body of a damaged neuron is infiltrated and bisected by branches of astrocyte that create a microscar around it while a microglial cell phagocytoses the damaged neuron. These coordinated actions are consistent with the scavenging and protective activities of microglia. In hypoxia, the neuron–astrocyte–microglia triads are more numerous in CA3 than in CA1, further indicating their protective effects. These data, taken from contiguous and interconnected hippocampal areas, demonstrate that glial response to the same hypoxic insult is not equal but varies significantly. Understanding the differences of glial reactivity is of great interest to explain the differential susceptibility of hippocampal areas to hypoxia/ischemia. Further studies may evidence the differential reactivity of glia in different brain areas, explaining the higher or lower sensitivity of these areas to different insults and whether glia may represent a target for future therapeutic interventions.

## Introduction

Neuroglial cells were discovered over a century ago and were first stained by the silver-chromate technique, characterized and drawn by Camillo Golgi in 1903. The concept of the physiological role of glial cells for substance exchange and metabolic support to neurons remains quite valid today. Activation of glia has been long considered solely detrimental for survival of neurons. Nevertheless, more recently it appears that the intercommunication among astrocytes–microglia and neurons is different not only in health and disease but can even modulate or control neurodegenerative mechanisms and may also vary depending upon the type of insult, and the different regions of the brain. No single-cell type in the brain is likely to be solely responsible for the etiopathogenesis of different neuropathological disorders such as Alzheimer’s disease (AD), Parkinson’s disease (PD), amyotrophic lateral sclerosis (ALS), traumatic brain injury (TBI), or brain ischemia. It is thus of the utmost importance to understand in deeper details what it means for glia to engage in phenotype switches and how they can influence surrounding cells. By becoming reactive, astrocytes and microglia undergo a set of transcriptional, functional, and morphological changes that transform them into cells with different properties and functions. Disruption of the finely tuned interplay between endothelial cells, pericytes, astrocyte end-feet contacts, microglia, oligodendrocytes, and neurons, all cells that form the neurovascular unit (NVU), likely contributes to the pathophysiology of neurodegeneration in chronic hypoxia and other neurodegenerative conditions such as AD or TBI. Indeed, the modified interplay among neurons and astrocytes/microglia may shift the balance from normal physiological conditions toward neurodegeneration (De Keyser et al., 2008; Sofroniew, 2009), but the precise role of astrocyte–microglia interactions with neurons in hypoxia-dependent mechanisms of neurodegeneration has not been clearly defined yet.

## Alterations of the Cerebrovascular Functionality in Chronic Hypoxia

Chronic cerebral hypoperfusion is one of the major mechanisms that cause the cognitive decline and dementia in aged patients. Chronic cerebral blood flow reduction is generally mild, with no sharp drop in the acute phase. Reduced cerebral perfusion correlates with the gravity of dementia and is a good predictor of who, among the patients with mild cognitive impairment (MCI), will later develop dementia (Gorelick et al., 2011). Clinical studies indicate that cerebrovascular pathologies are the primary causes of at least 20% of the cases of dementias and are cofactors in the pathogenesis of Alzheimer’s disease (AD) (Schneider et al., 2007; Gorelick et al., 2011; Toledo et al., 2013) and other neurodegenerative disorders such as Huntington disease (HD, Drouin-Ouellet et al., 2015) and ALS (Winkler et al., 2013). Stenosis or partial occlusion of the internal carotid arteries brings about reduction of cerebral blood flow (CBF) associated with chronic ischemia. These mild, chronic events result in impairment of memory and cognition in human patients, independently of the presence in the brain of severe lesions (Johnston et al., 2004; Marshall et al., 2012; Alosco et al., 2013; Balestrini et al., 2013; Stefansdottir et al., 2013). More intense reduction of CBF by about 40–50% causes suppression of brain activity and more profound cognitive dysfunctions, which are reversible after reestablishment of normal CBF (Tatemichi et al., 1995; Marshall et al., 1999, 2012). Even more intense, acute reductions of CBF cause ischemic stroke (Moskowitz et al., 2010), which doubles the risk for dementia. Around 30% of stroke patients develop cognitive dysfunction within 3 years (Leys et al., 2005; Pendlebury and Rothwell, 2009; Allan et al., 2011), and about 50% of patients younger than 50 years show cognitive deficits after a decade (Schaapsmeerders et al., 2013). Nowadays, the general consensus is that most cognitive impairments in the aged patients result from brain dysfunction caused by mild, chronic, cumulative damage to tissue and cells (Gorelick et al., 2011). Some published papers have revealed that the interplay between neurons and glial and vascular cells is particularly important in the prevention or development of vascular cognitive impairments (VCI) (Iadecola, 2010; Quaegebeur et al., 2011; Zlokovic, 2011). These data (Iadecola, 2010; Quaegebeur et al., 2011; Zlokovic, 2011) provide new avenues to reevaluate how modifications of cerebral blood vessels and of glia–neuron interaction contribute to neuronal dysfunctions and are responsible for cognitive impairment, calling for a reappraisal of the role of glial and cerebrovascular functionality in cognition.

The blood brain barrier (BBB) is disrupted in the course of chronic cerebral hypoperfusion (Rosenberg, 2012). Indeed, brain hypoxia is known to damage endothelial cells, pericytes, and astrocytes (Jiang et al., 2018) and cause increased leakage from the BBB (Al Ahmad et al., 2012), as demonstrated *in vivo* in a rat model of brain hypoperfusion produced by bilateral stenosis of the carotids (Ueno et al., 2002). Furthermore, breakdown of the BBB causes extravasation of plasma proteins, such as immunoglobulins, fibrinogen, and complement, all potent proinflammatory molecules, and increases the production of free radicals (Morgan et al., 1997; Yoshida et al., 2002; Davalos and Akassoglou, 2012; Crehan et al., 2013). Fibrinogen activates proinflammatory pathways that activate microglia and astrocytes (Davalos et al., 2012; Holland et al., 2015). Extravasation of proteins resulting from increased BBB permeability can exacerbate tissue edema, compressing blood vessels, reducing CBF, and increasing the hypoxic state of the tissue.

In addition, in rats and mice, cerebral hypoperfusion is associated with inflammation of the white matter and oxidative stress (Ihara et al., 2001; Masumura et al., 2001; Yoshizaki et al., 2008; Huang et al., 2010; Dong et al., 2011; Juma et al., 2011; Reimer et al., 2011). One of the main consequences of the oxidative and proinflammatory stress induced by hypoperfusion and consequent BBB breakdown is the damage to the myelin sheath. This causes modification of the integrity of the axons, demyelination, axonal loss, and decrease of the velocity of axon potential transmission (Franklin and Ffrench-Constant, 2008; Matute and Ransom, 2012). Axonal demyelination increases the requirements of energy of the denuded axons, aggravating the stress of the tissue. Indeed, oligodendrocytes are sensitive to increased levels of ATP and glutamate, which overactivate ionotropic glutamate receptors and P2X_7_ purinergic receptors (Bakiri et al., 2009; Verkhratsky et al., 2009). Overactivation of these receptors may kill oligodendrocytes by excitotoxicity (Arbeloa et al., 2012). These data may explain the studies in human patients that demonstrate a correlation between ischemic demyelination and stroke outcome (De Groot et al., 2002; Schmidt et al., 2003). All these effects amplify these pathogenic processes, exacerbating the damage to the brain tissue.

Furthermore, tissue hypoxia and oxidative stress activate transcription of many proinflammatory pathways through NF-kB, activating the expression of cytokines and adhesion molecules in vascular cells, reactive astrocytes, and activated microglia. Inflammation and oxidative stress have negative effects on the trophic interaction among the cells of the NVU. Reactive oxygen species (ROS) and inflammation suppress the prosurvival effects of endothelial cells on neurons by reducing BDNF levels (Guo et al., 2008).

## The Hippocampus

The hippocampus, a region of the brain fundamental for memory encoding, shows numerous structural, morphological, and electrophysiological alterations in many neurodegenerative disorders such as AD (Thal et al., 2002; Braak et al., 2006; Mueller et al., 2010; Small et al., 2011; Bartsch et al., 2015) and in ischemia (De Jong et al., 1999; Zola et al., 2000; Liu et al., 2005). All these alterations are at the basis of memory loss typical of advanced age, of ischemia, and of AD. The hippocampus forms a unidirectional network with the tri-synaptic pathway that originates *via* the perforant path from the entorhinal cortex (EC) and connects the dentate gyrus (DG) to CA3 and CA1 pyramidal neurons. CA3 neurons receive inputs from the DG *via* the mossy fibers and send axons to CA1 pyramidal cells *via* the Schaffer collateral pathway, as well as to CA1 in the contralateral hippocampus *via* the associational commissural pathway. In addition, CA1 neurons receive inputs directly from the EC *via* the perforant path and send axons to the subiculum. The hippocampal areas CA3 and CA1 have both morphological and anatomical similarities and differences. For instance, the pyramidal cell layer forms a continuum from CA3 to CA1 and have parallel inputs but have distinct network architectures and diverging output pathways (Amaral and Witter, 1989; Cenquizca and Swanson, 2007; Bahar et al., 2011). CA1 and CA3 subserve different functions and contribute to the processing of specific information such as novelty detection, encoding, short-term memory, intermediate-term memory, and retrieval (Vazdarjanova and Guzowski, 2004). CA1 is fundamental for mediating the association with temporal components and is capable of maintaining short-term memories (Wiebe et al., 1997), whereas CA3 is involved in processes associated with rapid formation of spatial or contextual memory (Lee and Kesner, 2002, 2003; Kesner et al., 2004; Nakazawa et al., 2003). CA3 and CA1 hippocampal areas, although interconnected through the Schaffer collaterals, and often considered as a continuum, respond differently to ischemic/hypoxic conditions (Kirino, 2000). Nevertheless, it is still not completely clear how and why these two contiguous, interconnected hippocampal areas respond in a different way to an ischemic event. In patients with cerebral hypoxia/ischemia, CA1 pyramidal neurons are among the most vulnerable (Zola-Morgan et al., 1986; Petito et al., 1987), as also repeatedly demonstrated in experimental animal models of hypoxia/ischemia (reviewed by Schmidt-Kastner and Freund, 1991) in the gerbil (Kirino, 1982), in the rat (Pulsinelli et al., 1982), and in humans (Bartsch et al., 2010, 2015). Indeed, since hippocampal CA1 circuits are fundamental for the processes of memory formation (Bartsch et al., 2010), impairment of CA1 neurons contributes to memory deficits in patients with damages to the hippocampus (Kadar et al., 1998).

The decrease of both oxygen (O_2_) and glucose supply caused by cerebral hypoperfusion gives rise to signaling failure in the vulnerable neurons of CA1 hippocampus, with impairment of hippocampally mediated learning and memory mechanisms (De Jong et al., 1999; Liu et al., 2005; Farkas et al., 2006; Melani et al., 2010; Lana et al., 2013). Many different hypotheses have been brought about to explain the higher sensitivity of CA1 neurons to deprivation of O_2_ and glucose caused by hypoxia or ischemia. O_2_ and glucose deprivation *in vitro* (OGD) is an established and widely used experimental model that allows exploring potential differences in the responses of rat hippocampal CA1 and CA3 neurons to ischemia (Gee et al., 2006; Pugliese et al., 2006; Cimarosti and Henley, 2008; Dixon et al., 2009; Sun et al., 2010). A few minutes after the beginning of OGD, deprivation of energy reduces the levels of intracellular ATP, with consequent failure of the Na^+^/K^+^ pump, anoxic depolarization, and increased extracellular levels of glutamate that is not taken up by the neuronal or astrocytic glutamate uptake system (Jabaudon et al., 2000; Rossi et al., 2000). Indeed, minutes after an ischemic insult, increase of extracellular glutamate is detected in both CA1 and CA3 areas (Mitani et al., 1992), leading in the gerbil to activation of glutamate receptors and neurotoxicity (Urban et al., 1990; Choi and Rothman, 1990). Glutamate NMDA receptor (NR) subunits play a fundamental role in Ca^2+^-induced excitotoxicity and are expressed differentially in the CA1 and CA3 hippocampal areas. NR2C is less permeable to Ca^2+^ ions than the other subunits (Chung et al., 2016). Interestingly, GluN2C knockout mice show greater neuronal death in the CA1 hippocampus a few hours after global cerebral ischemia (Chung et al., 2016). Furthermore, the balance between kinase and phosphatase activities in CA1 is in favor of tyrosine kinases, while in CA3 it is in favor of phosphatases (Gee et al., 2006). In the gerbil, ischemia increases tyrosine phosphorylation of NR2A and NR2B subunits of NMDA receptors (Zalewska et al., 2005). Furthermore, expression of NR2 receptor subtypes and splice variants is higher in CA1 than in CA3, explaining the higher vulnerability of CA1 after ischemia (Zola-Morgan et al., 1986; Wu et al., 2008). These data, taken together, may be one of the possible explanations of the higher sensitivity of CA1 to ischemia.

Neurons consume 75–80% of total brain energy (Hyder et al., 2013) for restoration of neuronal membrane potentials after depolarization (Harris et al., 2012), for neurotransmitter synthesis, vesicle packaging, axoplasmic transport, and neurotransmitter release (Attwell and Laughlin, 2001; Rangaraju et al., 2014; Pathak et al., 2015). Thus, energy demand in the brain is not uniform but is higher where neurons have higher neuronal activity. CA1 pyramidal neurons of the hippocampus have a higher firing rate than CA3 neurons and have higher energy demands (Mizuseki et al., 2012). For these reasons, it is therefore possible that CA1 neurons are preferentially vulnerable (Wilde et al., 1997; Padurariu et al., 2012) during an ischemic stress (De Jong et al., 1999; Wilde et al., 1997).

Deprivation of energy reduces the levels of intracellular ATP, modifies the ionic gradients, and inverts the glutamate uptake in rat pyramidal CA1 and CA3 neurons *in vitro* (Jabaudon et al., 2000; Rossi et al., 2000).

In addition, the vascular architecture of CA1 and CA3 areas show anatomical differences. While CA1 is vascularized by a large ventral artery, in CA3 many capillaries are present in the vicinity of neurons (Duvernoy et al., 1983). Indeed, increased neuronal activity is one of the major determinants of the dynamic increase of CBF to supply more blood, nutrients, and O_2_ to active neurons. Increased blood supply depends on the concerted action of vascular cells, astrocytes, and neurons (Iadecola, 2013). Many ions and vasoactive compounds with opposing effects, such as nitric oxide (NO), metabolites of arachidonic acid, adenosine, neurotransmitters, and neuropeptides (Drake and Iadecola, 2007), act as signals to regulate the hemodynamic changes of blood supply to a brain area. All these molecules are generated by synaptic activity of the afferents to the hippocampus from the basal forebrain and brainstem, by interneurons, and by astrocytes (Drake and Iadecola, 2007; Cauli and Hamel, 2010; Kleinfeld et al., 2011). These highly coordinated signaling pathways, with such a high degree of spatial precision and temporal definition (Iadecola, 2004), are probably necessary to fulfill the higher requirements of energy of CA1 in comparison to CA3 neurons in physiological conditions and may help in explaining the more intense sensitivity of CA1 to hypoxic/ischemic conditions. Indeed, it has been demonstrated (Chip et al., 2013) that a selective vascular vulnerability is present in CA1, which in turn is responsible for the depletion of blood supply to the CA1 subregions. Disruption of the BBB causes impairment of the astrocyte–vascular communication, with consequent modification of the BBB cytoarchitecture, alteration of regional homeostasis, and energy deficiency that leads to energy crisis (Alvarez et al., 2013). In case of increased energy demand, BBB disruption affects the functionality of energy-craving neurons, specifically those in CA1 that become unable to fulfill the high requirements of energy necessary for their functional homeostasis (Muddapu et al., 2020).

Furthermore, studies demonstrate that during ischemia the levels of superoxide and ROS are higher in the CA1 than in CA3 hippocampal area (Wilde et al., 1997; Wang et al., 2005) and induce stress-activated mitochondrial transition pores (MTPs) preferentially in CA1 than in CA3 rat hippocampus (Mattiasson et al., 2003). Activation of MTPs causes Ca^2+^-induced mitochondrial swelling that leads to microvacuolization (Duchen, 1992) and disruption of the mitochondria, consequent release of cytochrome C (CytC) in the cytoplasm, and apoptosis (Suen et al., 2008). Furthermore, in the Mongolian gerbil, post-ischemic mitochondrial damage is more severe in CA1 than in CA3 (Radenovic et al., 2011), indicating that CA1 and CA3 pyramidal neurons respond differently to similar stress conditions. Thus, CA1 pyramidal neurons seem more sensitive than CA3 neurons to the damage caused by production of ROS by mitochondrial and oxidative stress, as demonstrated in the aged mice (Kanak et al., 2013).

From all the data reported above, it appears that the real reason for the differential sensitivity of CA1 to hypoxia is not completely understood. Therefore, the study of the differences between CA1 and CA3 hippocampal areas is of fundamental importance, because it can explain the higher sensitivity of CA1 pyramidal neurons to different types of insults observed in both animal models of neurodegeneration and in patients (Mueller et al., 2010; Small et al., 2011; Bartsch and Wulff, 2015; Ugolini et al., 2018). On these bases, we compared the changes of the interplay among astrocytes–microglia and neurons as well as the modifications of neuroinflammatory markers in CA1 and CA3 hippocampus of rats in an *in vivo* model of cerebral hypoperfusion.

## The Neurovascular Unit and the Glymphatic System

The brain is dependent upon blood circulation in microvessels for delivering O_2_ and nutrients to neurons and for disposal of waste material. The NVU is composed by cells of different types and functions, all working synergistically in a highly regulated manner. Astrocyte endfeet surround the walls of the vessels, which, together with perivascular microglia and macrophages, survey the influx of molecules into the brain (Dudvarski Stankovic et al., 2016). In ischemic trauma, the disruption of blood vessels and BBB breakdown correlate with the accumulation of activated microglia, suggesting that microglia are associated with the dysfunction of blood vessels (Barkauskas et al., 2015). Disruption of the NVU is strongly associated with vascular dementia (Iadecola, 2013) and likely contributes to the early stages of AD, as shown in animal models of the disease (Iadecola et al., 1999; Iadecola, 2004; Kelly et al., 2017).

Furthermore, it has been demonstrated that in aged rats the decrease in the number of astrocytes is accompanied by decrease in VEGF expression, which further amplifies the vascular impairment (Bernal and Peterson, 2011). During ischemia, astrocytes are injured and show morphological changes such as swelling and vacuolization of the cell body and loss of their distal processes. All these modifications of astrocytes, named “clasmatodendrosis,” are caused by energy failure and acidosis (Friede and van Houten, 1961; Hulse et al., 2001). Clasmatodendrosis was first described by Cajal, as reported by Penfield (1928), and later rediscovered by Friede and van Houten (1961), and by Hulse and colleagues in hippocampal organ cultures (Hulse et al., 2001). Clasmatodendrosis is found in lesions of the white matter in patients with cerebrovascular disease and AD (Tomimoto et al., 1997). It is also present in the periventricular zone of patients with mixed dementia (Sahlas et al., 2002), in the corpus callosum of hypoperfused mice (Hase et al., 2017), in the hippocampus of rats with chronic epilepsy (Kim et al., 2011), and in aged rats (Cerbai et al., 2012). In AD and ischemia, clasmatodendrosis may represent an acute response of astrocytes to energy failure coupled with mitochondrial inhibition (Friede and van Houten, 1961; Kraig and Chesler, 1990; Hulse et al., 2001). Clasmatodendrotic morphological alterations of astrocytes are directly associated with changes in cell function (Jiang et al., 2018). Furthermore, an association between astrocyte injury and disruption of the BBB has been described in post-stroke surviving elderly patients (Chen et al., 2016). Disruption of the BBB can lead to inefficient removal and accumulation of toxins in the parenchyma, which may play a significant role in tissue damage. In subjects with MCI (Wang et al., 2006), increased permeability of the BBB has been observed at early stages of AD (Starr et al., 2009). All these findings support the idea that energy deficiency may be a cause of degeneration of neurons in ischemic conditions.

The channel aquaporin4 (AQP4), located on astrocyte endfeet, regulates the flux of water between blood and brain (Nagelhus and Ottersen, 2013) and is involved in regulation of BBB permeability (Tourdias et al., 2011). In mice, in the first few hours after ischemia, AQP4 is upregulated in the ischemic core, while the peak of its expression is found in the penumbra 48 h after ischemia (De Castro et al., 2006). Furthermore, mice deficient for AQP4 on astrocytes show significant reduction in water uptake and reduced brain edema following stroke, in comparison to wild-type animals (Haj-Yasein et al., 2011). After ischemia, reactive astrocytes increase the expression of connexin 43 and connexin 30 (Orellana et al., 2014), of AQP4 (Hirt et al., 2009; Hoshi et al., 2011), as well as of trophic factors such as BDNF (Neumann et al., 2015), which are molecules involved in neuron protection or in ischemic tolerance.

In the few hours after cerebral ischemia, hypoxia caused by the decrease of blood flow results in impairment of Na^+^/K^+^ ATPase, accumulation of intracellular Na^+^ which recalls water into the cell and induces cytotoxic edema (Simard et al., 2007). Astrocytes are the major cell type involved in cytotoxic edema (Kimelberg, 1995), and one of the key molecular players is AQP4 (Manley et al., 2000). The development of ischemic cellular damage causes breakdown of BBB, giving rise to leakage of plasma proteins to the extracellular space. Furthermore, swelling of astrocytes may compress the vessels in the ischemic regions, further decreasing the vessel caliber and exacerbating the hypoperfusion (Syková, 2001).

Since most of the high-affinity glutamate transporters are located on astrocyte membranes, astrocytes are the main cells involved in reuptake of glutamate at the NVU (Dallérac and Rouach, 2016). In the hippocampus, the two most expressed isoforms of glutamate transporters are excitatory amino acid transporter-1 (EAAT-1, GLAST in rodents) and excitatory amino acid transporter-2 (EAAT-2, GLT1) (Holmseth et al., 2012). In *in vivo* transient ischemia in the gerbil, astrocytic immunoreactivity for GLT1 is upregulated between 30 min and 12 hours after ischemia (Kim et al., 2006). In rat CA3 hippocampus, GLT1 immunoreactivity increases between 1 and 21 days after global ischemia (Bruhn et al., 2000). From these data, it can be postulated that increased uptake of glutamate and lactate may be the cause of astrocyte swelling the first hours/days after ischemia (Landis, 1994; Kimelberg, 2005; Verkhratsky et al., 2016). On the other hand, immunohistochemical studies show that GLT1 expression decreases in CA1 rat hippocampus between 2 and 4 days after ischemia reperfusion (Bruhn et al., 2000). Inconsistencies in these data may be due to differences of species or of brain regions.

Astrogliosis has been shown to be present in many neurodegenerative disorders, such as ischemia, AD, PD, ALS, and MS (for references, see Li et al., 2019). In addition, astrogliosis causes loss of AQP4 polarization in perivascular astrocytes, which potentially represents a mechanism common to NVU and glymphatic dysfunctions in many neurodegenerative diseases such as brain infarcts (Wang et al., 2017; Zbesko et al., 2018), AD (Kress et al., 2014; Xu et al., 2015), and TBI (Iliff et al., 2014; Guo et al., 2014; for references see also Rasmussen et al., 2018).

Nevertheless, reactive astrocytes seem to have both detrimental and beneficial roles (Sofroniew, 2009; Liddelow et al., 2017) in many neuropathological conditions such as ischemia (Pekny et al., 2019; Amantea et al., 2010; Barreto et al., 2011). The different responses of astrocytes to the ischemic insults depend, at least in part, on the severity of ischemia. In addition, other conditions such as age and spatial localization of astrocytes can determine whether astrocytic functions are protective or damaging, as will be discussed later. Nevertheless, in mice it has been demonstrated that activation of astrocytes is essential for induction of preconditioning, or ischemic tolerance, indicating the importance of reactive astrocytes for neuroprotection (Hirayama et al., 2015; for ref. see Koizumi et al., 2018).

As reported above, other data demonstrate that, due to metabolic stress, astrocytes located in CA1 have higher reaction to ROS, while the activity of glutamate transporters is reduced, leading to increased extracellular levels of glutamate that cause higher stress to CA1 neurons in rats after transient forebrain ischemia (Ouyang et al., 2007). Furthermore, astrocytes in pathological conditions express and release cytokines that disrupt the permeability of the BBB (Abbott, 2002). In addition, the clasmatodendrotic modifications of astrocytes that occur in ischemic conditions (Hulse et al., 2001) are further causes of BBB derangement. Disruption of BBB and of the glio-vascular network causes increase of endothelial cell permeability, reduction of glucose transport, and increased extracellular levels of toxic substances, which further increase neuroinflammation (Abbott, 2002; Zhu et al., 2007; Miyazaki et al., 2011; Freeman and Keller, 2012; Guan et al., 2013; Sweeney et al., 2018a, b).

In addition to the NVU, more recently the glia-lymphatic (glymphatic) system has come into focus as a highly specialized transport system that facilitates disposal of extracellular waste into the cervical and basal meningeal lymphatic networks or the dural sinuses. The glymphatic system consists of a network of perivascular or perineural channels supported by astrocytes (Reeves et al., 2020). Astrocytic endfeet express high levels of polarized AQP4 that facilitate not only the NVU but also the glymphatic flow, disposing of and clearing the interstitium from potentially toxic substances (Iliff et al., 2012). The glymphatic system is impaired during aging (Kress et al., 2014), and its dysfunction is involved in many neurodegenerative disorders, particularly those in which accumulation of extracellular waste such as Aβ and tau is an important pathogenetic mechanism, such as AD (Weller et al., 2009; Iliff et al., 2012; Jessen et al., 2015). Indeed, the glymphatic system represents a fundamental pathway in the net clearance of Aβ (Iliff et al., 2013; Xu et al., 2015). The clasmatodendrotic modification of astrocytes during aging (Cerbai et al., 2012) and ischemia (Hulse et al., 2001) may represent one of the causes not only of vascular but also of glymphatic dysfunction. Dysfunction of the glymphatic influx is secondary to acute ischemia, and multiple micro-infarction (Gaberel et al., 2014; Wang et al., 2017), but the astrocyte involvement on the efficiency of this system needs to be completely understood.

## Physiological and Pathological Actions of Astrocytes

Astrocytes, among the most represented glial cells in the central nervous system, have distinctive morphologies that differ both between and within regions of the brain. Astrocytes have many housekeeping functions, which help maintain a healthy brain (Verkhratsky and Nedergaard, 2018), Astrocytes control the formation, maturation, and plasticity of synapses by secreting thrombospondins, hevin, and solid-phase attachment of red cells (SPARC), all proteins that regulate synapse formation (Christopherson et al., 2005; Kucukdereli et al., 2011). Astrocytes control neural circuit formation through TNF-α (Stellwagen and Malenka, 2006) and TGF-β signaling (Diniz et al., 2012, 2014a, b, 2017). In addition, healthy astrocytes envelope synapses with their processes and are indispensable for neurotransmitter homeostasis, release of gliotransmitters, and maintenance and maturation of synapses (Pfrieger, 2009; Heneka et al., 2010). Furthermore, neuronal activity excites the membrane of astrocytes and, increasing intracellular Ca^2+^, induces the release of gliotransmitters (Perea and Araque, 2007; Navarrete et al., 2012; Araque et al., 2014), which are necessary for synaptic plasticity, indicating that astrocytes are involved in memory formation (Verkhratsky et al., 2011; Navarrete et al., 2012). In addition, astrocytes control the levels of the neurotransmitters GABA and glutamate at the synapses, thus mediating the synaptic functions (Sofroniew and Vinters, 2010) of the so-called tripartite synapse.

As already mentioned, healthy astrocytes maintain intimate contact with endothelial cells and pericytes through gap junctions, which allow intercellular diffusion of ions, regulate water and ion homeostasis, maintain the pH, allow the diffusion of small molecules, and contribute to functionality of the BBB (Siqueira et al., 2018). In this way, astrocytes provide energy required by neurons in the form of glucose (Rouach et al., 2008) and lactate (Figley, 2011; Sotelo-Hitschfeld et al., 2015), as well as trophic factors essential for neuronal survival (Nones et al., 2012; Dezonne et al., 2013). As reported above, astrocytes form an integral part of the BBB and of the glymphatic system and regulate neurovascular coupling, vascular tone, and blood flow (Sofroniew and Vinters, 2010; Macvicar and Newman, 2015; Verkhratsky and Nedergaard, 2018; Govindpani et al., 2019).

Astrocytes are maintained actively in a resting state, but the precise molecular signals that trigger astrocytic activation at the initial phases of an insult are still not known. Recent studies have demonstrated that different CNS injuries can stimulate at least two types of astrocytes with different properties, A2 reactive astrocytes that have beneficial, neuroprotective properties, and A1 reactive astrocytes that are harmful to neurons. A2 astrocytes are predicted to promote neuronal survival, outgrowth, synaptogenesis, and phagocytosis. On the contrary, A1 neuroinflammatory reactive astrocytes have harmful effects, upregulating many genes that express proinflammatory proteins and other factors that are destructive for synapses (for ref. see Liddelow and Barres, 2017). The “detrimental” A1 astrocytes, stimulated by inflammatory stimuli, upregulate genes associated with destruction of synapses and loss of neurons (Zamanian et al., 2012), while the “helpful” A2 astrocytes, induced by an ischemic event (Zamanian et al., 2012), upregulate cytokines such as TNFα. Inhibiting the proinflammatory cytokine IL-12p40 (Zakharova and Ziegler, 2005), TNFα has anti-inflammatory properties. A2 astrocytes upregulate neurotrophic factors and thrombospondins, predicted to stimulate development of synapses and survival of neurons (Zamanian et al., 2012).

A recent hypothesis postulates that in physiological conditions astrocytes may exist as a continuum of heterogeneous, mixed populations (Zhang and Barres, 2010; Khakh and Sofroniew, 2015; Ben Haim and Rowitch, 2016; Khakh and Deneen, 2019; Pestana et al., 2020). Consequently, the states of astrocytes vary in physiopathological conditions, depending not only on the type of insult but also possibly on the brain structure in which astrocytes are located (Liddelow and Barres, 2017). For instance, it is not clear yet whether different astrocytes located in different brain areas show the same morphofunctional modifications after a similar insult or whether astrocytes react differently to the same insult. The first hypothesis indicates that astrocytic responses are controlled by intrinsic cues, while the second hypothesis indicates that astrocytic responses are controlled by external, environmental signals (Martín-López et al., 2013; Bribian et al., 2018). Some recent insights give way to the idea that there may exist an apparent continuum in the intensity of astrocyte reactions to insults, which possibly hides different, discrete reactive states. Nevertheless, astrocyte reactivity differs among different areas of the brain. For instance, it has been demonstrated in both mice and humans that during aging astrocytes located in distinct brain regions have different transcriptional profiles (Soreq et al., 2017; Boisvert et al., 2018; Clarke et al., 2018) including glial fibrillary acidic protein (GFAP) and serpin (Boisvert et al., 2018) that result in complex region-specific molecular and morphological changes (Rodríguez et al., 2014). In AD, astrocytes become hypertrophic or atrophic depending not only on the stage of the disease but also to the proximity of Aβ plaques (Rodríguez-Arellano et al., 2016).

Recent evidences show that, although microglia represent the main phagocytic cells in the brain, astrocytes can participate in phagocytosis (Wu et al., 2009; Lu et al., 2011; Iram et al., 2016; Morizawa et al., 2017) after ischemia. Since so far astrocytes have received only limited attention as phagocytes, the mechanisms of astrocytic phagocytosis are still not completely understood. Nevertheless, it has been demonstrated that astrocytes use the ATP-binding cassette transporter (ABCA1) pathway (Morizawa et al., 2017), as well as multiple EGF-like-domains 10 (MEGF10) and proto-oncogene tyrosine-protein kinase MER (MERTK) pathways for phagocytosis (Chung et al., 2013). Microglia use the classical complement pathway to recognize and prune unwanted synapses in the developing mouse brain. Other studies suggest that astrocytes express other phagocytic receptors, such as brain-specific angiogenesis inhibitor 1 (BAI1) and integrin αvβ3 or αvβ5 (Park et al., 2007). Furthermore, in a transient middle cerebral artery occlusion mouse model, most of the phagocytic activity of astrocytes has a late onset after ischemia (7 days) and is localized mainly in the penumbra around the ischemic core (Morizawa et al., 2017), where neurons are slightly damaged and still recoverable. Most of phagocytic activity of microglia has an early onset after ischemia (1–3 days) and is located in the ischemic core where astrocytes are not phagocytic and where neurons and other cells are dead (Morizawa et al., 2017). Indeed, since astrocytes are not as mobile as microglia (Nimmerjahn et al., 2005; Okada et al., 2006), they are not able to migrate from the penumbra to the ischemic core. Astrocytes polarize their distal processes without cell body migration and engulf apoptotic bodies derived from dendrites of dying neurons, while microglia migrate toward damaged neurons and completely engulf dendrites, cell bodies, and nuclei (Damisah et al., 2020). Astrocytes located in the penumbra can become phagocytic and can contribute to clearance of debris and repair of the tissue (Morizawa et al., 2017). Furthermore, phagocytic microglia engulf larger debris than astrocytes (Morizawa et al., 2017), indicating that the size of debris that can be phagocytosed by astrocytes is limited (Damisah et al., 2020). Indeed, as will be discussed below, microglia can phagocytose live neurons during injury (Brown and Neher, 2014), inducing phagoptosis, a mechanism that contributes to neuronal cell death (Fricker et al., 2012; Neher et al., 2013). Astrocytes and microglia play specialized and orchestrated roles, acting in a highly coordinated fashion with spatiotemporal differences in different brain areas that can have important physiopathological consequences (Morizawa et al., 2017).

**FIGURE 1:**
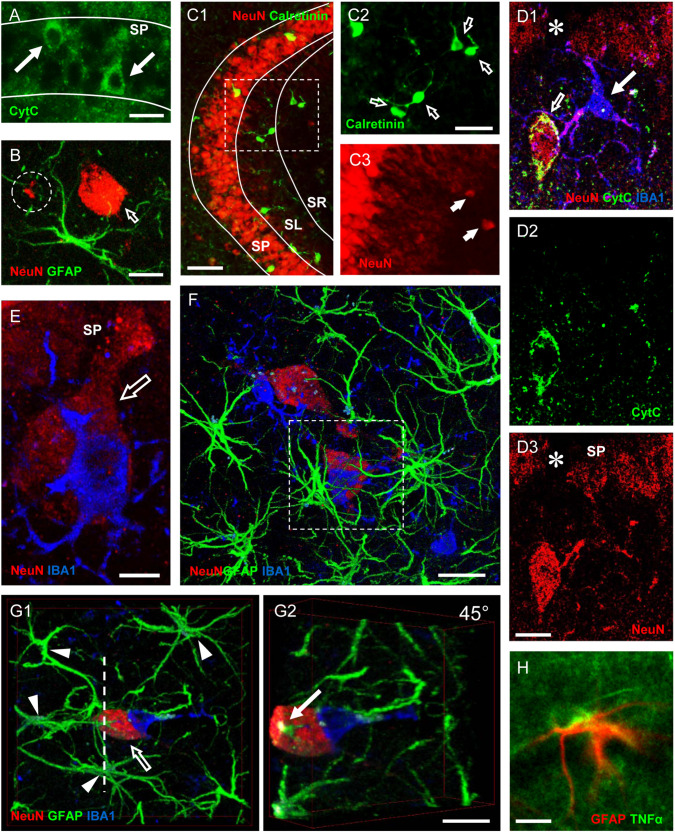
Characterization of neurodegeneration and alterations of the neuron–glia interplay in the bCCAo model of brain hypoperfusion in the rat. **(A)** Representative images showing CytC + apoptotic neurons in CA1 SP of a bCCAo rat (arrows). Scale bar: 15 μm. Adapted from Lana et al. (2014). **(B)** Representative image of a GFAP + astrocyte (green), a NeuN + damaged neuron (red, open arrow), and a NeuN + neuronal debris (in the circled area) in CA3 SR of a bCCAo rat. The neuronal debris appear closely apposed to the astrocyte branches. Scale bar: 10 μm. Adapted from Lana et al., 2017a. **(C1–C3)** Representative images showing NeuN + neurons (red) and calretinin + interneurons (green) in CA3 SP, SL, and SR of a bCCAo rat. Scale bar: 60 μm. **(C2–C3)** Magnifications of the framed area in **(C1)** showing the Calretinin + interneurons (open arrows, **C2**) and the NeuN + ectopic neurons (arrows, **C3**), demonstrating that ectopic neurons are not Calretinin+. Scale bar: 30 μm. Adapted from Lana et al., 2017a. **(D1–D3)** Representative images showing the colocalization of CytC (green) with NeuN (red) in the cytoplasm of an apoptotic–ectopic neuron (open arrow, **D1**) in the proximity of CA1 SP of a bCCAo rat. An IBA1 + microglial cell (blue, arrow, **D1**) projects its branches to surround the neuron. SP, appears indented in correspondence with the ectopic neuron (**D1,D3**, asterisk). Scale bar: 8 μm. Adapted from Lana et al. (2014). **(E)** Representative image showing a NeuN + ectopic neuron (open arrow) undergoing phagocytosis by an IBA1 + microglial cell (blue) in the proximity of CA1 SP of a bCCAo rat. The microglial cell resides on the top of the neuron and embraces it with its branches. Scale bar: 3 μm. Adapted from Lana et al. (2014). **(F)** Representative image showing two NeuN + ectopic neurons (red) surrounded by GFAP + astrocyte branches (green) and phagocytosed by IBA1 + microglial cells (blue) in CA1 SR of a bCCAo rat. In the framed area is shown a triad cluster. Scale bar: 10 μm. Adapted from Lana et al., 2014. **(G1–G2)** Representative image showing four GFAP + astrocytes (green, arrowheads) projecting their branches toward a NeuN + neuron (red, open arrow) undergoing phagocytosis by an IBA1 + microglial cells (blue) in CA1 SR of a bCCAo rat. Astrocyte branches form a glial “microscar” around the neuron. The digital subslicing of the neuron along the dotted line **(G1)** and the 45° rotation **(G2)** show that an astrocyte branch infiltrates the neuronal cell body (arrow, **G2**). Scale bar: 10 μm. Adapted from Lana et al. (2014). **(H)** Representative image showing a GFAP + TNFα + astrocyte (GFAP in red, TNFα in green) in CA3 SR of a bCCAo rat. Scale bar 15 μm. Adapted from Lana et al. (2017a).

In a less neuron-centric view of neurodegeneration, the alterations of astrocytes, such as decreased maintenance of brain homeostasis, impairment of buffering of extracellular glutamate, and reduced supply of nutrients to neurons, may contribute to the diffusion of damage to neurons and neurodegeneration (Miller et al., 2017). *In vitro* it has been shown that A1 astrocytes have a novel, deleterious function (Liddelow and Barres, 2017; Liddelow et al., 2017). A1 astrocytes secrete a neurotoxin that induces apoptosis in neurons and release toxic factors that target specifically motor neurons and mediate cell death in a mouse model of ALS (Re et al., 2014). We have first demonstrated in CA1 (Cerbai et al., 2012) and later in CA3 (Lana et al., 2016), as well as in DG (Lana et al., 2017b) of hypoperfused rats, that astrocytes send branches to embrace, infiltrate, and bisect apoptotic neurons (Figures 1G1,G2). This mechanism is finalized to the fragmentation of apoptotic, dying neurons to form cellular debris in order to spare and protect the surrounding tissue from the damage caused by the release of proinflammatory products in the parenchyma. Ours are the first demonstrations that astrocyte branches infiltrate the cytoplasm of apoptotic neurons, to bisect the dying neuron and form debris. We have demonstrated that neuronal debris are indeed more numerous in the CA1 and CA3 stratum radiatum (SR) of hypoperfused rats than in controls, and neuronal debris are all closely apposed to the branches of astrocytes (Figure 1B) and ready to be phagocytosed by microglia (Figure 1E). While adaptive, reactive astrogliosis has been shown to have beneficial effects, suppression of astrocyte reactivity may also increase neuronal vulnerability, exacerbating the pathology and altering regeneration (Burda and Sofroniew, 2014; Pekny et al., 2014). In addition, it is possible that when astrocyte reactivity becomes too intense, the release of neurotoxic factors, such as components of the complement cascade that enhance synaptic degeneration (Stevens et al., 2007; Hong et al., 2016; Sekar et al., 2016), and of neurotoxins that cause the death of motor neurons (Di Giorgio et al., 2007; Nagai et al., 2007; Lobsiger et al., 2007) could be responsible for increased neurotoxicity. Thus, astrocytes can behave as actors in causing or preventing neurodegeneration.

The comprehension of the multiple, contrasting roles of astrocytes in the pathophysiological mechanisms of neurodegeneration, a theme that only recently entered into focus, will be of great interest in understanding the pathogenesis of many neurodegenerative disorders such as AD, PD, ALS, and TBI.

## Physiological and Pathological Actions of Microglia

Microglia represent 5–10% of the brain cells and are the resident immune cells of the central nervous system (Frost and Schafer, 2016). In physiological conditions, the highly mobile processes of microglia dynamically reorganize by cyclically forming and withdrawing (Nimmerjahn et al., 2005), thus allowing microglia to patrol the brain parenchyma (Nimmerjahn et al., 2005). Under physiological circumstances, microglial cells in resting state have small somata and fine, ramified branches. Upon detection of proinflammatory stimuli, microglial cells activate rapidly and become major actors of the neuroinflammatory response (Crotti and Glass, 2015; Hickman et al., 2018). Microglial cells that rapidly undergo morphological and genetic changes upon activation are first responders to insults such as ischemic brain injury (Kettenmann et al., 2011). Disruption of brain homeostasis causes morphofunctional changes such as cell body hypertrophy and thickening of the branches. Contemporarily, many cell surface markers are upregulated, such as cluster of differentiation (CD) 45, major histocompatibility complex II, and CD68 (Ransohoff and Perry, 2009). Activated microglia have dual roles in the ischemic brain, depending on the stimulus. Microglia can acquire a spectrum of different but overlapping functional phenotypes, including the classical pro-inflammatory and the anti-inflammatory alternatively activated phenotypes. Microglia produce a plethora of cytokines and chemokines that promote inflammatory mechanisms, BBB dysregulation, and leukocyte infiltration (da Fonseca et al., 2014). Activated microglia may also have beneficial effects, phagocytosing cellular debris, and suppressing inflammatory responses, as reported by Neumann and coworkers (Neumann et al., 2006) in a model of ischemia in rat organotypic hippocampal slices *in vitro*. The dual roles of microglia may depend on their phenotypic polarization after ischemia (Franco and Fernández-Suárez, 2015; Hu et al., 2015; Jiang et al., 2016; Xiong et al., 2016). Microglial cells patrol the parenchyma to detect and eliminate debris or apoptotic neurons by phagocytosis (Hsieh et al., 2009; Lana et al., 2017a). It has been shown by 2-photon imaging in the zebrafish spinal cord (Morsch et al., 2015) and in mouse brain (Davalos et al., 2005; Nimmerjahn et al., 2005) that microglia exhibit ramified processes with high motility that allow a dynamic and continual survey of brain parenchyma and have an active role in the surveillance and maintenance of healthy brain. Indeed, activation of microglia is now viewed as a multistage, reversible process that generates multiple phenotypes of reactive cells with protective abilities (Hanisch and Kettenmann, 2007; Ransohoff and Perry, 2009; Kettenmann et al., 2013). Microglial projections are chemotactic sensors that extend toward injured cells in the “find-me” step of neuron phagocytosis (Hanisch and Kettenmann, 2007). Therefore, damage of microglial projections may weaken the neuroprotective activity of microglia. Reasonably, decreased microglial migration may hamper its phagocytic efficacy, favoring the accumulation of degenerating neurons and proinflammatory neuronal toxic debris (Tian et al., 2012), typical of brain aging (Cerbai et al., 2012). Nevertheless, it is still accepted that chronic, exaggerated activation of microglia, such as in chronic inflammatory diseases, can cause robust pathological alterations and neurobehavioral complications (for references see also Glass et al., 2010; Norden and Godbout, 2013). Recent work from Barres’s lab demonstrates that during inflammatory responses activated microglia modify their secretory profile, increase the release of factors such as C1q, TNF-α, IL-1α, and influence astrocyte activation (Liddelow et al., 2017). Nevertheless, microglial activation and production of cytokines have positive effects in early brain development (for ref. see Salter and Beggs, 2014), in synaptic pruning (for references see Schafer and Stevens, 2013), and in normal learning and memory in mice (Ziv et al., 2006; Derecki et al., 2010).

It is now known that two types of activated microglia exist, characterized by different phenotypes (Allen and Barres, 2009) and opposed effects. The proinflammatory M1 state occurs when microglial cells are activated by an acute insult and release proinflammatory mediators such as NO, ROS, quinolinic acid, and cytokines such as TNFα, IL-1, IL-6, and IL-18. The non-inflammatory, repairing M2 state of microglia, associated with secretion of anti-inflammatory cytokines such IL-4, IL-10, IL-13, and TNF-ß, also has a role in tissue repair. The role of microglia in neurodegeneration depends on the expression of apolipoprotein E (APOE) and triggering receptor expressed on myeloid cells 2 (TREM2) (Krasemann et al., 2017). In acute models of neurodegeneration, APOE regulates TREM2, which in turn modulates the activation of microglia (Krasemann et al., 2017).

Nevertheless, as for A1 and A2 astrocytes, this quite recent classification of microglia in M1 and M2 states (Liddelow and Barres, 2017) seems to be rather narrow, not corresponding to the variety of microglia phenotypes so far discovered in the brain. The activation profile of microglial cells is not an all or none response but can be imagined as a continuous, highly dynamic process that depends on the type of insult and is also influenced by the area(s) of the brain, the stimuli, and disease progression (De Biase et al., 2017; Keren-Shaul et al., 2017). It can also change during the progression of the pathological conditions. Indeed, the distinction between the protective or pathogenic roles of activated microglia could depend on the timing, and/or severity of insult, and/or on the location of the microglia cell. According to Halder and Milner (2019), in the mouse spinal cord during the early phase of vascular damage and leakage, microglia mediate a protective response to maintain vascular integrity. When the damage is continued, microglia may become inappropriately stimulated into a phagocytic phenotype. Indeed, in the early stages of a model of ischemic stroke in the mouse, microglia congregate around damaged blood vessels, but later microglia switch from a repair phenotype into a phagocytic one, removing the dying endothelial cells (Jolivel et al., 2015). Furthermore, in a mouse model of cerebral ischemia, decreased proliferation of microglia leads to increased neuronal death and larger lesion after stroke, indicating a protective role of microglia (Wolf et al., 2017).

Furthermore, regional differences may play an important role in the resting status and responsiveness to insults. Indeed, in a study it has been demonstrated in the mouse that the microglial transcriptome and their sensitivity and response to insults vary in a region-dependent way (Grabert et al., 2016). Furthermore, microglia express many different receptors such as the purinergic P2, toll-like, TNF-α, fractalkine R1 (CX3CR1), and TREM2 receptors, among many others (Mariani and Kielian, 2009; Gelderblom et al., 2015; Hug et al., 2018). All these receptors have a variety of functions, and their expression and the outcome of their activation depend on the functional state of the cell and on the pathological conditions. Depending on the nature of the ligand and on the receptor, downstream intracellular pathways translate their activation to detrimental or beneficial effects (for references see Gomes-Leal, 2012). For instance, ATP binds to different purinergic receptors with beneficial or detrimental effect after ischemia (Gelderblom et al., 2015). ATP, activating its receptors on microglia, stimulates the inflammasome, which causes increased secretion of proinflammatory cytokines that spread and intensify the inflammatory environment. ATP binds to purinergic P2 receptors and is involved in the formation or resolution of inflammation (Eltzschig et al., 2012). Among the P2 purinergic receptors, the metabotropic G-protein-coupled P2Y receptor (P2YR) and the nucleotide-gated ion channel P2X receptor (P2XR) are the most studied. Within the P2YR family, P2Y2R and P2Y6R both promote the phagocytic clearance of apoptotic cells or bacteria, contributing to the termination of inflammation (Koizumi et al., 2007). On the contrary, microglial P2Y12R seems to be involved in mediating damage in cerebral ischemia (Webster et al., 2013). The P2 × 7R likely participates in the cerebral damage associated with stroke (Domercq et al., 2010).

In preclinical studies, increased density and activation of microglia following hypoperfusion (Reimer et al., 2011; Manso et al., 2018) is associated with release of matrix metalloproteinase-2 (MMP-2) (Simpson et al., 1999). MMPs are proteases that degrade the extracellular matrix (ECM) and the tight junctions between endothelial cells. Furthermore, MMPs are involved in BBB breakdown after ischemia (Seo et al., 2013) and can degrade myelin (Chandler et al., 1995). Increased production of ROS by activated microglia may disrupt NO signaling, causing endothelial dysfunction (Freeman and Keller, 2012). Thus, microglia may further damage the BBB through a proinflammatory cascade that causes degradation of the ECM by production of MMPs and by oxidative damage.

Age is a key risk factor for vascular cognitive impairment and dementia (VCID) and is associated with modifications of phenotype and functions of microglia (von Bernhardi et al., 2015), which assume a more intense proinflammatory phenotype (Norden and Godbout, 2013). Aging microglia have less branchings, reduced motility, and lower migration rates that confer an altered surveillance phenotype and have a more sustained inflammatory response to damage (Damani et al., 2011). The phenotypic modifications seem to be caused by a shift in microglial metabolic pathways since activated microglial cells treated with an inflammatory stimulus show a reduction in mitochondrial oxidative phosphorylation (Nair et al., 2019). Minocycline is a potent inhibitor of inflammatory responses that has preclinical efficacy in several animal models of VCID and particularly in vascular conditions in which microglial cells are activated, including cerebral hypoperfusion (Ma et al., 2015; Manso et al., 2018). Chronic administration of minocycline reduces the number of microglial cells, restores the hypoperfusion-induced impairment in white matter function, and has protective effects (Ma et al., 2015; Manso et al., 2018).

Apoptosis is a mechanism of controlled cell death and may subserve a homeostatic function to maintain and regulate the number of cells in health and pathological conditions (Kerr et al., 1972; Becker and Bonni, 2004). Increased apoptosis is thought to be a physiological mechanism that helps maintain normal tissue homeostasis through resolution of low-grade inflammation, such as those that develop during normal aging (Gupta et al., 2006). Therefore, in the chronic, low-grade inflammatory conditions typical of chronic brain hypoperfusion, brain parenchyma surveillance by astrocytes and patrolling by microglia seem to be aimed at reducing the spreading of inflammation from apoptotic neurons and debris, preventing further damage to neighboring neurons. As a consequence, under these conditions, astrocytes and microglia have a protective role, cooperating in the disposal of neuronal debris by phagocytosis or of whole neurons by phagoptosis (Lana et al., 2017a), clearing dysfunctional synapses, controlling proinflammatory mediators, and diffusing damage to neighboring cells. How apoptosis causes neurons to be disposed of is still uncertain. The principal mechanism is probably by triggering the release of intercellular signals, such as the “find-me” signals ATP and fractalkine (CX3CL1) (Noda et al., 2011; Cerbai et al., 2012) and the “eat-me” signals such as phosphatidylserine (PS) (reviewed in Márquez-Ropero et al., 2020), which recall and activate phagocytic cells such as microglia to engulf and consume the neuron. The release of “don’t eat me” signals from neurons, such as CD47-SIRPα or CD200-CD200L, maintains microglia in a quiescent state and suppresses phagocytosis (reviewed in Michell-Robinson et al., 2015). Astrocytes and microglia express membrane receptors, such as CX3CR1, P2Y6, P2Y12, stabilin 1, SIRPα, TREM2, MerTK, and CD11b (Takahashi et al., 2005; Peri and Nüsslein-Volhard, 2008; Wakselman et al., 2008; Mazaheri et al., 2014), that recognize molecules released by damaged neurons (Harrison et al., 1998; Noda et al., 2011), causing the phagocytosis of degenerating neurons and neuronal debris. Nevertheless, in a model of hypoperfusion in the rat, no significant decrease of pyramidal neurons in both CA1 and CA3 (Cerbai et al., 2012; Lana et al., 2016) was found. The damaged neurons are possibly replaced by the continuous addition of newborn neurons by neurogenesis (see below).

## Metabolism of Astrocytes and Microglia During Hypoperfusion

The brain is an energetically demanding organ, and most of energy supply, in form of ATP, is utilized by neurons to meet the high energy requirements necessary for neuronal activity such as the synthesis and release of neurotransmitters and neuromodulators and for the maintenance of the ionic gradients necessary for synaptic activity (Attwell and Laughlin, 2001). In astrocytes, energy stores are localized mainly as glycogen that represents a short-term buffer for transient energy requirements from neurons (Kong et al., 2002) that do not store energy. Brain metabolic requests and modifications strongly influence the origin and progression of many neurodegenerative disorders such as AD and PD (for references see Aldana, 2019). Nevertheless, an important fraction of energy in the brain is not directly requested by neurons for their activity. Normal brain activity mainly depends on metabolic plasticity of astrocytes and requires not only glucose supply from blood but also glycogen stored in astrocytes that fuels specific activity in the brain and that can last beyond the limits of glucose supply from blood (Brown and Ransom, 2007). Glycogen in astrocytes is essential for the survival of axons, and its depletion is related to brain dysfunctions and neurodegeneration (Wender et al., 2000). Noradrenaline and insulin regulate glycolysis and glycogenesis in astrocytes, while ATP production in the mitochondria and oxidation of fatty acids is regulated by the thyroid hormone (Morita et al., 2019). In response to inflammation or oxidative stress, astrocytes upregulate glycolysis producing ATP and lactate, which support energy metabolism to neurons but also accelerate neurodegeneration by fueling reactive astrocytes (Almeida et al., 2001). It appears therefore that the energy metabolism of reactive astrocytes is a determinant of physiological processes, and its impairments or modifications may cause significant decline of brain functions (Morita et al., 2019). It has also been shown that in hypoxic/ischemic conditions the neuroprotective A2 reactive astrocytes upregulate fatty acid oxidation (FAO) (Zamanian et al., 2012), which is neuroprotective. Upregulation of fatty acid metabolism attenuates inflammation by clearing fatty acid from the nearby parenchyma (Takahashi et al., 2014). The ketone bodies, produced by fatty acid oxidation in astrocytes, maintain the energy metabolism in neurons, compensating for the glucose metabolism damaged by ischemia and by production of ROS (Takahashi et al., 2014).

Microglia, for their role in brain development, for their continuous scanning of brain parenchyma to maintain a healthy environment, and for their harmful responses to injuries and activation of repair programs (Aldana, 2019; Engl and Attwell, 2015), have large energy demands. Furthermore, during insults and brain tissue damage, microglia are the first responders to pathological changes to homeostasis, migrate to the site of injury, modify their morphology retracting their processes, and phagocytose debris and cells (Davalos et al., 2005). It is not completely understood whether microglial motility and phagocytosis are powered by oxidative phosphorylation or by glycolytic pathways, but either one or both these processes represent a significant energy demand (Engl and Attwell, 2015).

Recent findings demonstrate that peripheral immune cells can adapt to environmental challenges, modifying their metabolic pathways in order to utilize nutrients other than glucose, such as fatty acids or amino acids (Van den Bossche et al., 2017). Recently, it has been demonstrated (Bernier et al., 2020) in models of hypoglycemia or aglycemia that activities of microglia, such as process motility and damage sensing functions, are maintained by alternative metabolic pathways such as glutaminolysis, which depend on mammalian target of rapamycin (mTOR) activation. This metabolic plasticity sustains mitochondrial metabolism even in brain neuroenergetic crisis, allowing microglia to maintain their fundamental surveillance and phagocytic roles (Bernier et al., 2020). Furthermore, in aged mice, microglia display changes in metabolic profile such as modifications of proteins involved not only in inflammatory signaling but also in mitochondrial function and cellular metabolism (Flowers et al., 2017). Indeed, oxidative stress is associated with non-pathological aging, and functional decline of mitochondria in microglia increases the production of ROS and inflammatory mediators that in turn may increase oxidative stress.

Therefore, it appears that perturbation in the regulation of brain energy metabolism in neurons, as well as in astrocytes and microglia, may be one of the pathophysiological mechanisms of neurodegenerative disorders among which AD, PD, ALS, and HD, and this can be even more important in hypoxia/ischemia-dependent pathologies. Indeed, metabolic disturbances such as high blood pressure, atherosclerosis, obesity, and diabetes are among the most important risk factors for dementia (Kivipelto et al., 2006), as shown by epidemiological studies. Deficits of energy metabolism such as hypometabolism of glucose and mitochondrial dysfunctions in both neurons and astrocytes are early indicators of neurodegenerative disorders such as AD, PD, ALS, and HD (Hoyer, 1993; Diehl-Schmid et al., 2007; Edison et al., 2013; Adiele and Adiele, 2019).

## Neuron–Astrocyte–Microglia Interactions in the Hippocampus in a Rat Model of Chronic Hypoperfusion

Brain chronic hypoperfusion is a progressive, dynamic process induced by partial carotid occlusion during aging, heart failure, hypotension, atherosclerosis of large or small vessels, and carotid stenosis. Brain chronic hypoperfusion causes multiple progressive modifications that eventually lead to vascular dementia and neurodegeneration (Chmayssani et al., 2007; Farkas et al., 2007; Ozacmak et al., 2007), which may manifest with cognitive dysfunctions.

The rat model of brain chronic hypoperfusion, obtained with the permanent bilateral occlusion of the common carotid arteries (bCCAo) (Sarti et al., 2002a, b; Farkas et al., 2007; Lana et al., 2014, 2017a), represents a model of cerebrovascular stenosis in aging humans. This model is helpful in investigating the mechanisms and effects of long-term chronic cerebral hypoperfusion (Farkas et al., 2007). bCCAo in the rat causes chronic cerebral hypoperfusion mainly in the forebrain and leads to early disruption of the BBB, to white matter rarefaction with axonal and myelin damage, to neuroinflammation, and to hippocampal and cortical neuronal damage (Ciacciarelli et al., 2020). Indeed, the infarcts generated by bCCAo are seen not only in the striatum and the dorsolateral cortex but also in areas such as the hippocampus, thalamus, and hypothalamus (Ciacciarelli et al., 2020). Among the plausible explanations for the unexpected brain damages to the latter brain areas are (i) anomalies of the circle of Willis (Kitagawa et al., 1998), (ii) production of neurotoxic molecules that propagate to the hippocampus and cause ischemic damage (Xie et al., 2011), and (iii) overexcitation of the hippocampal neuronal network by glutamate produced after the occlusion. In addition, an alternative explanation is that small and deep arteries beyond the circle of Willis, such as the anterior choroidal artery (AchA), the lateral hypothalamic artery (LHA), and the ventral thalamic artery (VTA), have a function in the supply of blood to deep brain areas. The AchA provides the major blood supply to the anterior hippocampus and other deep areas. In addition, these blood vessels can originate directly from the internal carotid artery, proximally to the origin of middle cerebral artery (MCA). Therefore, whereas distal occlusions cause lesions to the striatum, the piriform cortex, and portions of the parietal–temporal cortex, proximal occlusions may produce damage to the anterior hippocampus, thalamus, and/or hypothalamus.

In our investigations in the rat model of bCCAo (Lana et al., 2014, 2017a), we highlighted and characterized the quantitative, morphological, and functional alterations on neuron, astrocyte, and microglial interactions that may be relevant for the neurodegenerative processes in CA1 and CA3 hippocampus. Our findings demonstrate the existence of common and differential features of the interplay among neurons and glia in the two hippocampal areas, which may help in explaining the higher sensitivity of pyramidal neurons in CA1 to hypoxia leading to neurodegeneration. We developed the innovative *ex vivo* method of the triple-labeling fluorescent immunohistochemistry coupled with confocal microscopy (TIC) and digital imaging, which we exploited to make comparisons between areas CA1 and CA3 of the hippocampus. Using this novel method, we also implemented the new technique of digital subslicing (Lana et al., 2014, 2017a), which allows to visualize the intimate interplay among different cells, and the colocalization of different antigens that can take place within the cell. With this novel method, we studied the morphological and functional alterations of the neuron–astrocyte–microglia triad as a possible mechanism responsible on one side for neuroprotection and on the other one for the neurodegeneration that characterizes animal models of neurodegenerative diseases such as AD or ischemia (Lana et al., 2014, 2017a; Fusco et al., 2018; Ugolini et al., 2018).

In CA1 and CA3 str. pyramidalis (SP) of hypoperfused rats, numerous apoptotic neurons are present, characterized by intense and uniform CytC immunostaining in the cytoplasm (Figure 1A), a hallmark of the late phases of apoptosis (Suen et al., 2008). In both CA1 and CA3 str. radiatum (SR) of hypoperfused rats in the proximity of SP, we found the presence of numerous neurons with pyramidal shape, the so-called ectopic neurons. More specifically, in area CA3 the ectopic neurons are localized mainly in str. lucidum (SL) (Figures 1C1–C3), which is an “a-neuronal” region of CA3, as paradigmatically defined by Amaral and Lavenex (2007). Ectopic neurons are not interneurons (see Figures 1C1–C3) but likely are degenerating pyramidal neurons that derive from SP and are being detached from it. Indeed, ectopic neurons infiltrated by astrocyte branches (see Figures 1G1,G2), are located in close proximity to SP, which appears indented in correspondence with the ectopic neuron (Figures 1D1,D3, asterisk). We hypothesize that apoptotic CA1 neurons are removed from CA1 SP, possibly by astrocyte branches through signaling molecules such as the Cx43 and/or CX3CL1 (Noda et al., 2011; Cerbai et al., 2012). The ectopic neurons detached from the pyramidal layer in CA1 and CA3 are apoptotic and undergo the process of phagocytosis by microglia (Figures 1D1–D3). Furthermore, as a consequence of apoptosis, ectopic neurons are fragmented to form neuronal debris, present in high density in both CA1 and CA3 SR of hypoperfused rats (circled area in Figure 1B). This is possibly a protective mechanism that avoids the diffusion of inflammatory damage to surrounding neurons in response to proinflammatory substances released in the parenchyma.

As demonstrated in other models of neurodegeneration, astrocyte branches, infiltrating the neuronal cell body, may trigger or accelerate the fragmentation of apoptotic neurons (Polazzi and Monti, 2010; Cerbai et al., 2012; Huizinga et al., 2012). Neuronal debris are located throughout SR of CA1 and CA3, not in a random position but mostly closely apposed to astrocyte branches (circled area in Figure 1B). This phenomenon is similar to that observed in normal brain aging and LPS-induced neuroinflammation (Cerbai et al., 2012; Lana et al., 2016, 2017b).

We postulate that the detachment of a neuron from the pyramidal layer to form ectopic neurons that are fragmented into neuronal debris is part of a common mechanism of neuronal death in the hippocampus in a milieu of hypoxia/ischemia, in acute or chronic inflammatory conditions that can lead to neurodegeneration.

Despite all the above mechanisms, in hypoperfused rats, the quantity of CA1 and CA3 pyramidal neurons does not decrease (Lana et al., 2014, 2017a). Increased neurogenesis (Farkas et al., 2007; Dirnagl, 2012; Maraula et al., 2013) during the restitution phase of brain chronic hypoperfusion can explain this apparently contradictory result. Newborn neurons migrating from the subgranular zone of the DG to CA1 and CA3 pyramidal layers possibly reintegrate the apoptotic neurons.

Numerous phagocytic events by microglia on the ectopic neurons detached from the pyramidal layer (Figure 1E) also involve astrocytes that form peculiar clusters of cells with neurons and microglia that we define “triads.” In a triad, one or more astrocyte(s) project its branches toward a neuron that is phagocytosed by a microglial cell (Figures 1F,G1). According to our findings, the branches of astrocytes involved in the triad exert a fundamental role. They form a protective microscar around the neuron that is undergoing phagocytosis, to prevent the spread in the surrounding tissue of proinflammatory mediators and of neuronal debris, which could initiate an inflammatory response potentially harmful for other healthy neurons. Astrocyte branches exert a noxious effect on the neuron that is involved in the triad: they can infiltrate the neuronal body to help or accelerate its fragmentation (see Figures 1G1,G2). The mechanism of infiltration and fragmentation of the neuronal cell body by astrocytes branches, first demonstrated in our laboratory in models of normal brain aging and acute inflammation (Cerbai et al., 2012), was later confirmed in a model of ALS (Re et al., 2014) and in other papers published by our group (Lana et al., 2014, 2016). The triad formation and the astrocyte-mediated fragmentation of the ectopic-apoptotic neurons to form debris (Cerbai et al., 2012; Lana et al., 2014, 2016, 2017a, b) could be a common mechanism of neuronal death in a neurodegenerative milieu in the hippocampus. We also hypothesize that this could be a general mechanism of neuronal death also valid in healthy tissue for senescent neurons. In the hippocampus of hypoperfused rats, the expression of TNF-α increases in astrocytes and dendrites of pyramidal neurons in both CA1 and CA3 SR (Figure 1H). Microglia and astrocytes can both recognize danger signals, including those released by cellular debris produced from apoptotic cells, and can cooperate and help clearing apoptotic neurons or neuronal debris (Medzhitov and Janeway, 2002; Milligan and Watkins, 2009). This concerted action can prevent or reduce release of proinflammatory mediators and consequent injury to neighboring neurons (Nguyen et al., 2002; Turrin and Rivest, 2006).

Some events reflect a common response of CA1 and CA3 to the hypoperfusion, but the two areas show different responses to the ischemic insult. In particular, these differences are related mainly to behavior of glial cells, confirming their primary function in influencing the pathophysiology of the brain. In CA1 SR of hypoperfused rats, astrocytes show no modifications (Lana et al., 2014), whereas in CA3 SR, astrocytes significantly increase in response to hypoperfusion but do not appear hypertrophic or hyperactivated (Lana et al., 2017a). Higher demand of O_2_ and nutrient supply by pyramidal neurons that are in a hypoxic and hypoglycemic state due to hypoperfusion can possibly cause the increase of astrocyte density, which can balance the reduced trophic support to CA3 neurons. This response of astrocytes, contrary to that observed in CA1, is possibly a protective effect of astrocytes toward neurons.

Astrogliosis, a late-emerging event during chronic cerebral hypoperfusion (Pappas et al., 1996; Farkas et al., 2004, 2006, 2007; Schmidt-Kastner et al., 2005), has long been considered a negative phenomenon. This idea is rapidly changing, thanks to new data that suggest a more elaborated and diversified role of astrocytes upon different insults leading to neurodegenerative disorders such as AD, ALS, and stroke (Sofroniew, 2009; Verkhratsky et al., 2013; Burda and Sofroniew, 2014). Dysfunctions in the reactivity of astrocytes can be the primary cause, or can contribute to loss of normal functions of neurons, leading to or increasing neurodegeneration (Sofroniew, 2009).

Another remarkable difference is the behavior of microglia which decreases in CA1 (Lana et al., 2014) and increases in CA3 SR of hypoperfused rats (Lana et al., 2017a). These differences may explain the higher sensitivity of CA1 pyramidal cells to an ischemic insult. Recruitment and activation of microglia to the site of the insult generally is believed a negative mechanism that causes accumulation of neurotoxic phagocytes. Recently, microglial activation following neuronal injury is considered a reversible multistep process that represents mainly a protective mechanism (Minghetti and Levi, 1998; Streit et al., 1999; Polazzi and Contestabile, 2002; Hanisch and Kettenmann, 2007; Ransohoff and Perry, 2009; Kettenmann et al., 2013). In microglia-depleted organotypic cultures, CA1 pyramidal cell death increases, suggesting a neuroprotective role of microglia (Montero et al., 2009). Apoptotic neurons can release diffusible signals that enhance microglia neuroprotective properties. In turn, microglia release molecules that can rescue neurons from apoptosis (Polazzi et al., 2001). Phagocytosis of apoptotic cells by microglia decreases the production of pro-inflammatory cytokines, such as TNF-α and IL-12, without affecting the secretion of anti-inflammatory, potentially neuroprotective molecules, such as IL-10 and TGF-ßl (Magnus et al., 2001). During ischemia, microglia are responsible for the phenomenon of phagoptosis (also called primary phagocytosis), first defined by Brown and Neher (2012) as “death caused by being devoured.” Phagoptosis represents the phagocytosis of whole neurons that show no sign of neurodegeneration (Zhang et al., 2015). It is triggered by a stressful stimulus which is too mild to cause cell death, too intense to allow restoration of the healthy neuron, but sufficient to release “find-me” signals that activate and recruit microglia and astrocytes for phagocytosis (Ravichandran, 2011; Kao et al., 2011; Brown and Neher, 2012).

Thus, formation of triads appears as a specific mechanism for clearance of neurons under degeneration, not only through the mechanism of phagocytosis but also through phagoptosis (Brown and Neher, 2014). The increase of microglia that help in the disposal of damaged neurons (Nathan and Ding, 2010) may result in anti-inflammatory and neuroprotective effects (Liesz et al., 2009). Increased microglia in area CA3 of hypoperfused rats during the restitution phase (Farkas et al., 2007; Dirnagl, 2012) may release anti-inflammatory cytokines that trigger an anti-inflammatory milieu (Spite and Serhan, 2010). Microglia in CA3 SR (Beynon and Walker, 2012) phagocytose apoptotic pyramidal neurons or neuronal debris, promoting tissue repair and the resolution of inflammation. Taken together, our results demonstrate that effects of astrocytes and microglia in pathological conditions may contribute to neuronal damage but may also be a mechanism of protection to control the proinflammatory process and the diffusion of the cellular damage to the surrounding cells.

## Conclusion

The data of this review contribute to deepening the concept that the interactions that occur among the different cell populations of the CNS give rise to reciprocal networks of morphological and functional reliance and dependency. To comprehend the peculiar aspects of the onset and progression of neurodegeneration, it is necessary to consider that any tissue, and mainly the nervous tissue, is not composed by a collection of independent elements but rather by interdependent cell populations that interact and cooperate to maintain the homeostasis and functionality of the organ. Different types of insults that affect one population modifying its functionality reasonably reverberate to the others, either favoring or dysregulating their activities. In the neuron–astrocyte–microglia triads, the cell body of a damaged neuron is infiltrated and bisected by astrocyte branches that form a microscar around it and is embraced by microglia cells for the purpose of phagocytosing it. While activation of glia has long been considered as a detrimental mechanism for neuron survival, recently it is emerging that this is not always the case. Nevertheless, in contiguous, interconnected hippocampal areas, the responses of glia to the same insult are not equal but vary significantly from CA1 area to CA3.

**FIGURE 2:**
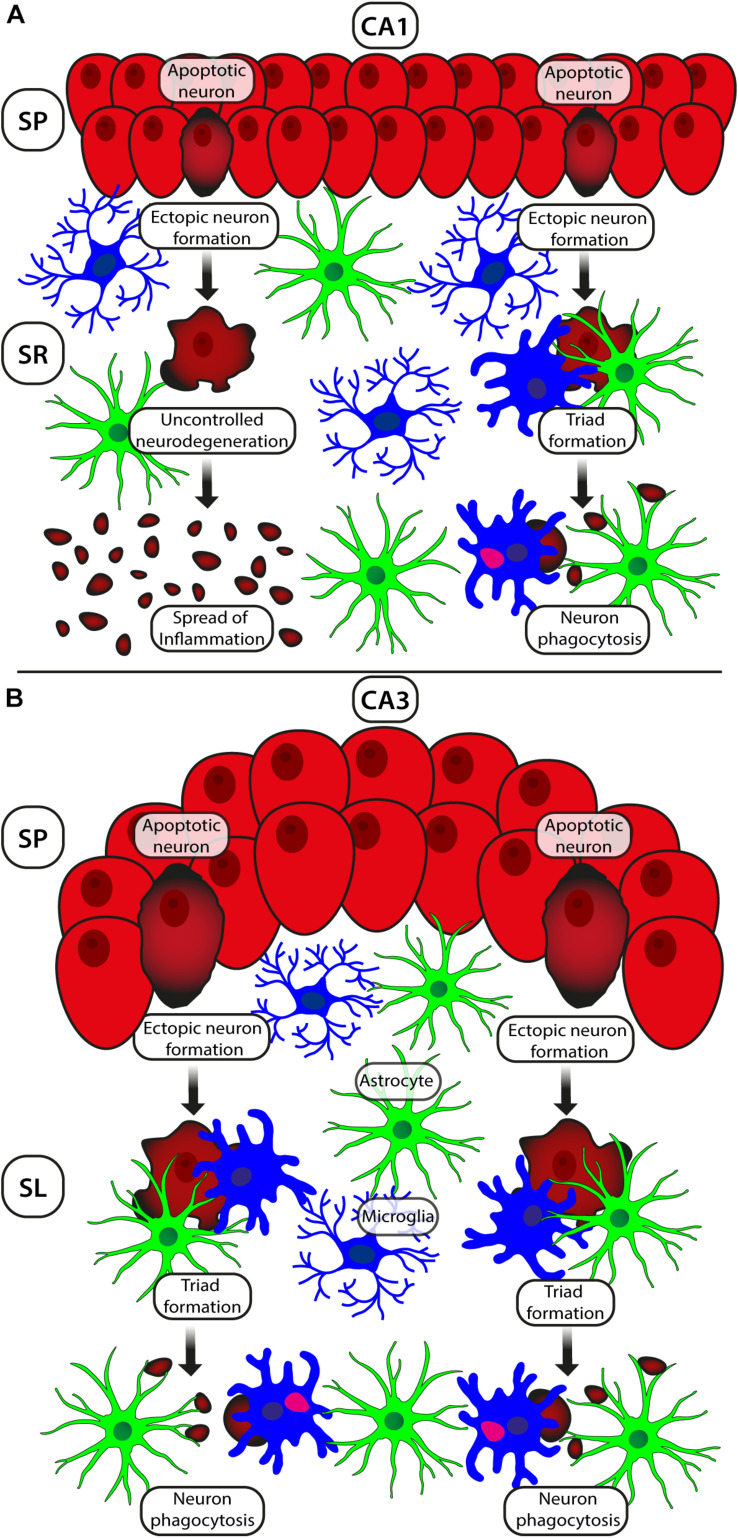
Schematic representation of the different responses of neurons and astrocytes–microglia to ischemia/hypoxia in areas CA1 **(A)** and CA3 **(B)**. The increase of apoptotic neurons (shown in dark red) is comparable in the two regions, but the density of ectopic pyramidal neurons in CA3 (dark red) is higher than in CA1. Pyramidal apoptotic neurons translocate from their competence layer to the underlying SR (CA1) or SL (CA3) to form triad clusters, be fragmented by astrocytes and be phagocytosed by microglia. This mechanism is finalized to the protection of the surrounding neurons in CA1 SP. In CA3 of ischemic rats, microglia and triads are significantly more numerous than in CA1, a further sign of better responsiveness of CA3 to hypoxia. Astrocytes are more numerous in CA3, possibly because of the increased trophic request of the tissue. This effect may be a further mechanism of a better response of CA3 to the ischemic insult. In CA3, astrocytes and microglia may help control the inflammatory process and the ensuing diffusion of the cellular damage to the surrounding tissue.

The comparison between the response of areas CA1 and CA3, shown schematically in Figure 2, helps to understand the differential reactivities of the two areas in hypoxia and to understand why pyramidal neurons in CA3 show higher adaptation than those in CA1 to hypoxia/ischemia (Lana et al., 2014, 2017a). The increase of apoptotic neurons is similar in the two regions, but in CA3 the density of ectopic pyramidal neurons is significantly higher than in CA1. The translocation of neurons from their competence, pyramidal layer to the contiguous SL or SR allows the damaged neurons to be fragmented by astrocytes to form neuronal debris that can be phagocytosed by microglial cells in the triad clusters. All these mechanisms are protective effect toward the surrounding neurons. In addition, the migration of neurons from the SP can decrease the diffusion of toxic/proinflammatory compounds to neighboring vital neurons. Likely, in CA3 the scavenging mechanism on degenerating pyramidal neurons is more active, explaining the lower sensitivity of the hippocampal CA3 area to the ischemic insult. Indeed, in CA3 of ischemic rats microglia is significantly higher than in the CA1 area where microglia decrease. As mentioned earlier, since activation of microglia is considered a protective mechanism (Hanisch and Kettenmann, 2007; Ransohoff and Perry, 2009; Kettenmann et al., 2013), the increase of microglia in CA3 may explain the better response of this area to an ischemic insult, in comparison to CA1. Lastly, in CA3 there is a significant increase of astrocytes, a phenomenon that can depend on the increased trophic request of the tissue and may represent a sign of a better response of CA3 to the ischemic insult. Finally, the neuron–astrocyte–microglial triads are significantly more numerous in CA3 than in CA1 of ischemic rats, further indicating the protective effects of the triads. Taken together, these results demonstrate that in CA3 astrocytes and microglia interplay with apoptotic neurons may represent a protective mechanism able to control the inflammatory process and the diffusion of the cellular damage to the neighboring tissues.

It is of great interest to verify if the differences of glial reactivity in these two contiguous hippocampal areas are mirrored in many other areas of the brain, reflecting the higher or lower sensitivity to different insults, and whether they may represent targets for future therapeutic interventions.

## Author Contributions

MG and DL wrote the manuscript. FU contributed to the preparation of the figures. MG edited and revised the manuscript. All authors read and approved the final manuscript.

## Conflict of Interest

The authors declare that the research was conducted in the absence of any commercial or financial relationships that could be construed as a potential conflict of interest.

## Abbreviations

ABCA1: ATP-binding cassette transporter
AD: Alzheimer’s disease
ALS: Amyotrophic lateral sclerosis
APOE: Apolipoprotein E
BAI1: Brain-specific angiogenesis inhibitor 1
bCCAo: Bilateral occlusion of the common carotid arteries
BDNF: Brain-derived neurotrophic factor
CBF: Cerebral blood flow
CNS: Central nervous system
CX3CL1: Fractalkine
CX3CR1: Fractalkine receptor1
Cx43: Connexin43
CytC: Cytochrome C
FAO: Fatty acid oxidation
GFAP: Glial fibrillary acidic protein
GLAST, Excitatory amino acid transporter-1: EAAT-1
GLT1, Excitatory amino acid transporter-2: EAAT-2
HD: Huntington’s disease
IBA1: Ionized calcium binding adaptor molecule 1
LPS: Lipopolysaccharide
MCI: Mild cognitive impairment
MEGF10: Multiple EGF-like-domains 10
MERTK: Proto-oncogene tyrosine-protein kinase MER
MHC: Major histocompatibility complex
MMP: Matrix metalproteinases
MS: Multiple sclerosis
mTOR: Mammalian target of rapamycin
MTP: Mitochondrial transition pore
NeuN: Neuronal nuclei
NF-kB: Nuclear factor-kappa B
OGD: Oxygen and glucose deprivation
PD: Parkinson’s disease
ROS: Reactive oxygen species
SL: Stratum lucidum
SP: Stratum pyramidalis
SPARC: solid-phase attachment of red cells
SR: Stratum radiatum
TBI: Traumatic brain injury
TGF- β: Transforming growth factor-beta
TIC: Triple-labeling fluorescent immunohistochemistry coupled with confocal microscopy
TNF- α: Tumor necrosis factor- α
TNF: Tumor necrosis factor
VCI: Vascular cognitive impairments
VCID: Vascular cognitive impairment and dementia
VEGF: Vascular endothelial growth factor.
